# Sex differences in healthcare expenditures among adults with diabetes: evidence from the medical expenditure panel survey, 2002–2011

**DOI:** 10.1186/s12913-017-2178-3

**Published:** 2017-04-11

**Authors:** Joni S. Williams, Kinfe Bishu, Clara E. Dismuke, Leonard E. Egede

**Affiliations:** 1 Center for Patient Care and Outcomes Research, Medical College of Wisconsin, 8701 Watertown Plank Road, Milwaukee, WI 53226 USA; 2 Department of Medicine, Division of General Internal Medicine, Medical College of Wisconsin, 9200 W. Wisconsin Ave, Milwaukee, WI 53226 USA; 3 Center for Health Disparities Research, Department of Medicine, Medical University of South Carolina, 135 Rutledge Avenue, Room 280, MSC 250593, Charleston, SC 29425 USA; 4 Department of Medicine, Division of General Internal Medicine and Geriatrics, Medical University of South Carolina, 171 Ashley Avenue, Charleston, SC 29425 USA; 5 Health Equity and Rural Outreach Innovation Center/Center of Innovation (HEROIC/COIN), Ralph H. Johnson Department of Veterans Affairs Medical Center, 109 Bee Street, Mail Code 151, Charleston, SC 29401 USA

**Keywords:** Sex differences, Healthcare expenditures, Adults, Diabetes

## Abstract

**Background:**

The evidence assessing differences in medical costs between men and women with diabetes living in the United States is sparse; however, evidence suggests women generally have higher healthcare expenditures compared to men. Since little is known about these differences, the aim of this study was to assess differences in out-of-pocket (OOP) and total healthcare expenditures among adults with diabetes.

**Methods:**

Data were used from 20,442 adults (≥18 years of age) with diabetes from the 2002–2011 Medical Expenditure Panel Survey. Dependent variables were OOP and total direct expenditures for multiple health services (prescription, office-based, inpatient, outpatient, emergency, dental, home healthcare, and other services). The independent variable was sex. Covariates included sociodemographic characteristics, comorbid conditions, and time. Sample demographics were summarized. Mean OOP and total direct expenditures for health services by sex status were analyzed. Regression models were performed to assess incremental costs of healthcare expenditures by sex among adults with diabetes.

**Results:**

Fifty-six percent of the sample was composed of women. Unadjusted mean OOP costs were higher for women for prescriptions ($1177; 95% CI $1117–$1237 vs. $959; 95% CI $918–$1000; *p* < 0.001) compared to men. Unadjusted mean total direct expenditures were also higher for women for prescriptions ($3797; 95% CI $3660–$3934 vs. $3334; 95% CI $3208–$3460; *p* < 0.001) and home healthcare ($752; 95% CI $646–$858 vs. $397; 95% CI $332–$462; *p* < 0.001). When adjusting for covariates, higher OOP and total direct costs persisted for women for prescription services (OOP: $156; 95% CI $87–$225; *p* < 0.001 and total: $184; 95% CI $50–$318; *p* = 0.007). Women also paid > $50 OOP for office-based visits (*p* < 0.001) and > $55 total expenditures for home healthcare (*p* = 0.041) compared to men after adjustments.

**Conclusions:**

Our findings show women with diabetes have higher OOP and total direct expenditures compared to men. Additional research is needed to investigate this disparity between men and women and to understand the associated drivers and clinical implications. Policy recommendations are warranted to minimize the higher burden of costs for women with diabetes.

## Background

Diabetes is the seventh leading cause of death, accounting for significant morbidity and mortality and 20% of total health care dollars in the United States [1]. The American Diabetes Association (ADA) approximates total costs for diabetes care in the United States to be around $245 billion secondary to high indirect and direct medical costs, lost productivity, and increased disability [2, 3]. In the United States, individuals with diabetes have average medical expenditures of nearly $8000 for diabetes care alone, a cost 2.3 times higher than costs for individuals without diabetes [2–4]. Recent evidence estimates total direct expenditures for individuals with diabetes in the United States to be about $218.6 billion unadjusted and $46 billion adjusted per year [4]. When diagnosed earlier in life (i.e., 40 years old), the excess lifetime medical spending for people with diabetes in the United States has been estimated at $124,600 per person when discounted (and approximately $211,400 per person without any discounts) compared to individuals without diabetes [1]. Ultimately, as the prevalence of diabetes in America continues to increase and the population continues to age, overall healthcare expenditures, including out-of-pocket (OOP) costs, associated with diabetes management will continue to rise, unless prevention is emphasized and the risk for complications reduced [1, 2, 5].

Evidence shows that, from the time of diagnosis, expenditures associated with diabetes care occur as a result of inpatient hospitalizations (43%), prescription medications for comorbid conditions and diabetes-related complications (18%), diabetes medications and supplies (12%), physician office-based visits (9%), and stays at skilled nursing and residential care facilities (8%) [2, 6, 7]. Between 1993 and 2006 alone, more than a 65% increase in discharges from inpatient hospitalizations were reported for patients with diabetes [8]. According to data from the National Ambulatory Medical Care Survey (NAMCS) and National Hospital Ambulatory Medical Care Survey (NHAMCS), adult patients (≥18 years of age) with diabetes made approximately 291,922,000 visits to the offices of primary care providers or hospital outpatient clinics for care between 2006 and 2010 [9]. Given the intricate nature of diabetes management, which often results in higher rates of healthcare utilization and services, it is no surprise that costs associated with diabetes care are high for adults with diabetes and continue to escalate.

For optimal diabetes management, patients with diabetes need comprehensive care, which can involve multiple and various types of healthcare services, resulting in increased OOP and overall expenses. This is especially true for non-Hispanic Blacks and women, who have been shown to have higher total health care expenses for diabetes compared to other racial and ethnic groups and men, respectively [1–3]. Generally, women tend to have significantly higher healthcare expenditures and use more healthcare services than men [10–12]. This is believed to be due, in part, to a higher rate of chronic conditions such as diabetes, hypertension, and obesity, in women compared to men [6, 13]. Women, compared to men, often report multiple comorbid chronic conditions [14], which leads to more prescribed medications, increased numbers of provider visits, and ultimately, higher healthcare costs [6, 13–15]. Evidence suggests this trend remains true between women [15] and men diagnosed with diabetes [1]; however, the evidence is sparse. Therefore, given little is known about these differences and the need for additional evidence, the aim of this study was to assess sex differences in OOP and total healthcare expenditures among adults with diabetes, with the hypothesis that women with diabetes will have higher expenditures compared to men with diabetes.

## Methods

### Data source and study population

Cross-sectional data for calendar years 2002–2011 from the Medical Expenditure Panel Survey Household Component (MEPS-HC) were combined together to serve as a “pooled” sample and used to examine the association between healthcare expenditure and sex among adults with diabetes (aged ≥18 years). All measures are based on previously validated questionnaires that are publicly available on the MEPS website [16–18]. In this retrospective study, we identified 20,442 (weighted sample of 17,820,243) adults with self-reported diabetes from MEPS-HC, who were alive during the calendar year.

MEPS is a compilation of large-scale surveys from the United States civilian, non-institutionalized population that includes data on families and individuals and their medical providers and employers [16–18]. It is a complete source of data on the cost and use of healthcare and health insurance coverage by families and individuals and is designed to obtain annual use and expenditure data for at least two calendar years for a selected sample of households [16–18]. To collect MEPS data, computer-assisted personal interviewing is conducted annually, with interviews lasting approximately 90 min [16–18]. Administered by the Agency for Healthcare Research and Quality (AHRQ) [16–18], MEPS is a self-reported instrument validated by the AHRQ using a variety of quality assurance procedures.

To ensure sufficient sample size and robust estimation for our analysis, we pooled (i.e., combined) 10 years of MEPS data to identify trends over time. Given commonalities in the variance structure of the variables, we confirmed compatibility and comparatively of the variables within the complex sample design. To estimate the nationally representative sample of the U.S. population, our study accounts for the sampling weights, clustering, and stratification design of the MEPS data [18]. Finally, MEPS data from adults with type 1 and type 2 diabetes are included in this study, as no distinction between the type of diabetes can be determined given the design of the MEPS data.

### Measures

#### Variables of interest

The dependent variables in this study were OOP expense and total direct expenditure and their components including prescribed medicine expenditures, office-based medical provider expenditures, hospital inpatient expenditures, hospital outpatient expenditures, emergency department expenditures, dental expenditures, home healthcare expenditures and other expenditures. In MEPS, total direct expenditures are the direct payments for care made during the year, including OOP expenses and payments by private insurance, Medicaid, Medicare, and other sources. Out-of-pocket expenses comprise only the portion of the total direct expenditures made by individuals and families for services received during the year. The primary independent variable was sex, which was dichotomized as female versus male. Using the Consumer Price Index obtained from the Bureau of Labor Statistics, the 2002–2011 costs were adjusted to 2014 dollar value [19].

### Controlled covariates

Racial/Ethnic groups were categorized into: Non-Hispanic White (NHW), Non-Hispanic Black (NHB), Hispanic, or others. Education was classified as: less than high school (≤ grade 11), high school (grade 12), and college or more (grade ≥ 13). Marital status was grouped into: married, non-married, and never married. Age was categorized into: 18–44, 45–64, and ≥ 65 years. Metropolitan Statistical Area (MSA) was coded by MSA status (MSA = 1, Non-MSA = 0). Census region was compartmentalized as: Northeast, Midwest, South, and West. Health insurance was categorized into: private only, public only, and uninsured at all times throughout the year. The income level was defined as a percentage of the poverty level and grouped into four categories: poor (<125%), low income (125% to less than 200%), middle income (200% to less than 400%), and high income (≥400%). Because of their association with diabetes and their influence on medical expenditures, comorbid conditions including hypertension, cardiovascular disease, stroke, emphysema, joint pain, arthritis, and asthma were included in the analyses as binary indicators [20, 21]. Binary indicators of comorbidities were based on a positive response to the question “have you ever been diagnosed with “hypertension, cardiovascular disease (CVD) (which included coronary heart disease, angina, myocardial infarction, or other heart diseases), stroke, emphysema, joint pain, arthritis and asthma.” Previous studies showed that the binary indicators of disease are more effective in accounting for disease burden [22, 23]. For the pooled data sample, calendar year was grouped into five consecutive years of 2002/03, 2004/05, 2006/07, 2008/09 and 2010/11. All of the covariates controlled for in the analyses were based on self-report.

### Analyses

The sociodemographic characteristics of adults with diabetes were presented by sex status, with percentage differences for categorical variables tested using *χ* ^2^ tests. The unadjusted mean direct medical expenditures were estimated for individuals by sex status using test post estimation survey command.

A two-part model-first, a probit model to approximate positive medical expenditure compared to zero expenditure, and second, a generalized linear model (GLM) to calculate the adjusted total medical expenditures based on a positive medical expenditures-was used to estimate the adjusted annual medical spending [24–28], especially given the ability of a two-part model to accommodate circumstances with excessive zeros such as the use of expenditure data [26, 29]. The use of GLM in the second part of the model (instead of log Ordinary Least Squares, or OLS), was advantageous since it relaxes the normality and homoscedasticity assumptions of the data and avoids bias associated with retransforming to the raw scale [26]. The Park test was used to examine the fit of the model; it verified the use of a gamma distribution with a log link as the best–fitting GLM for consistent estimation of coefficients and marginal effects of medical expenditures [28, 30]. The Variance Inflation Factor (VIF) indicated no multicollinearity problems existed between predictors of the two-part model. To control for confounding, sociodemographic factors including age, race, marital status, education, health insurance, MSA, region, income level, and comorbidities were included in the final model. All analyses were performed at the person-level using Stata 14 [31].

## Results

Table 1 shows a summary of the sample demographics for adults with diabetes (≥18 years of age) in this study. The unweighted sample size was 20,442 for adults with diabetes, which represented a weighted sample of 17,820,243 within the U.S. population. Of the unweighted sample, 44% identified as male and 56% as female. Approximately 47% of the sample was between the ages of 45 and 64, while 40% was 65 years of age and older. Sixty-four percent of the sample was NHW, followed by 15% NHB, 14% Hispanics, and 7% of other racial and ethnic backgrounds. Fifty-nine percent was married, and 74% had a high school diploma or higher. Sixty-one percent had private insurance, and the majority (80%) lived in an urban area (i.e., MSA). Forty percent of the sample was from the South, with approximately 20% representing the Midwest and West regions. Approximately one-third of the sample rated their income as either middle or high as associated with the federal poverty line. Finally, statistically significant differences in age, race/ethnicity, marital status, educational level, insurance coverage, income, and comorbid conditions including cardiovascular disease, joint pain, arthritis, and asthma were observed between men and women in the sample.

**Table 1:** Weighted sample demographics by sex among adults with diabetes

Variables	All (%)	Men (%)	Women (%)	*P*-value
N (n)	17,820,243 (20,442)	8,692,709 (9032)	9,127,534 (11,410)	
Age category				
Age 18–44	13.5	12.5	14.5	<0.001*
Age 45–64	46.8	49.4	44.4	
Age 65–85	39.7	38.1	41.1	
Race/ethnicity				
Non-Hispanic White	64.3	67.9	60.9	<0.001*
Non-Hispanic Black	15.4	12.6	18.1	
Hispanic	13.5	12.7	14.2	
Others	6.8	6.8	6.8	
Marital status				
Married	58.6	69.8	47.9	<0.001*
Non-married	32.2	21.8	42.1	
Never married	9.2	8.4	10.0	
Education category				
< High School	26.0	23.8	28.2	<0.001*
High School	34.4	32.1	36.5	
College or more	39.6	44.1	35.3	
Insurance				
Private	60.9	66.1	56.0	<0.001*
Public	31.4	26.0	36.5	
Uninsured	7.7	7.9	7.5	
Metropolitan statistical status				
Urban	79.8	79.2	80.4	0.138
Rural	20.2	20.8	19.6	
Census region				
Northeast	18.1	17.6	18.6	0.293
Midwest	21.0	21.0	21.1	
South	40.1	39.8	40.4	
West	20.8	21.6	19.9	
Income category				
Poor income	20.1	15.6	24.4	<0.001*
Low income	16.3	14.1	18.3	
Middle income	30.4	30.6	30.2	
High income	33.2	39.7	27.1	
Chronic conditions				
Hypertension	72.9	72.1	73.6	0.172
CVD	31.7	33.5	29.9	<0.001*
Stroke	10.2	10.1	10.3	0.839
Emphysema	4.9	5.1	4.7	0.346
Joint pain	55.7	50.7	60.5	<0.001*
Arthritis	48.5	39.8	56.7	<0.001*
Asthma	13.7	9.1	18.1	<0.001*
Year category				
Year 2002/03	15.5	15.4	15.6	0.559
Year 2004/05	18.0	17.6	18.3	
Year 2006/07	20.3	20.4	20.2	
Year 2008/09	22.7	22.5	22.9	
Year 2010/11	23.5	24.1	23.0	

*N* Weighted sample size, *n* Unweighted sample size, *%* Weighted percentage

*Statistically significant at *p* < 0.05

Table 2 shows the unadjusted mean of health services for out-of-pocket expenses and total direct expenditures by sex among adults with diabetes. The OOP costs for the pooled sample was statistically different with women spending $1878 compared to men spending $1631 (95% Confidence Interval (CI) $1788–$1969 vs. $1544–$1717, respectively; *p* < 0.001). For specific health services, women were found to have higher OOP expenses and total direct expenditures compared to men. Statistically significant differences were observed in OOP expenses for prescriptions with women having approximately $218 more OOP expenses in a given year compared to men ($959; 95% CI $918–$1000 vs. $1177; 95% CI $1117–$1237; *p* < 0.001). Similarly, statistical differences in prescription costs were observed between men and women for total direct expenditures. In a given year, prescription costs for women totaled approximately $3797 (95% CI $3660–$3934) compared to $3334 (95% CI $3208–$3460) for men (*p* < 0.001). In addition, women had higher total direct expenditures within a year for home healthcare compared to men ($752; 95% CI $646–$858 vs. $397; 95% CI $332–$462; *p* < 0.001).

**Table 2:** Unadjusted mean of health services for expenditures by sex among adults with diabetes

	Mean, Men ($)	95% CI	Mean, Women, ($)	95% CI	*P* value
Out-of-pocket expenses					
Prescription	959	918–1000	1177	1117–1237	<0.001***
Office-Based	225	200–250	244	223–265	0.263
Inpatient	89	43–135	77	44–110	0.679
Outpatient	42	34–50	49	41–57	0.217
ER Visit	26	15–38	18	15–22	0.195
Dental	182	156–207	167	141–193	0.420
Home Healthcare	23	8–38	44	23–64	0.092
Others	81	69–93	99	84–114	0.059
Pooled sample	1631	1544–1717	1878	1788–1969	<0.001***
Total direct expenditures					
Prescription	3334	3208–3460	3797	3660–3934	<0.001***
Office-Based	2340	2156–2523	2372	2246–2498	0.768
Inpatient	3790	3402–4179	3660	3327–3993	0.611
Outpatient	954	822–1085	963	834–1093	0.919
ER Visit	294	262–326	295	264–326	0.967
Dental	334	301–368	312	277–346	0.344
Home Healthcare	397	332–462	752	646–858	<0.001***
Others	199	178–220	206	179–233	0.662
Pooled sample	11,646	11,080–12,212	12,361	11,839–12,882	0.055

*Level of significance *p* < 0.05; ** level of significance *p* < 0.01; ***level of significance *p* < 0.001

*Abbreviations*: 95% CI = 95% Confidence Interval. Reported as dollars in 2014

Table 3 shows the adjusted incremental effects of healthcare expenditures by sex among adults with diabetes. For OOP expenses, women had higher costs within a given year for prescriptions ($156; 95% CI $87–$225; *p* < 0.001), office-based visits ($53; 95% CI $24–$83; *p* < 0.001), and other healthcare expenses ($19; $7-$30; *p* < 0.001) compared to men. The pooled sample paid approximately $242 (95% CI $134–$350; *p* < 0.001) OOP for healthcare services. Similar to observations for OOP expenses, women paid approximately $184 (95% CI $50–$318; *p* = 0.007) in total direct expenditures for prescriptions. They also spent more in total direct expenditures for home health services ($59; 95% CI $2–$116; *p* = 0.041). A statistically significant difference in total direct expenditures was not observed in the pooled sample; however, it was found among the pooled sample for OOP expenses, which suggests different factors may be driving OOP expenses in women compared to men that are causing them to have higher costs.

**Table 3:** Two-part regression model-Incremental effects of healthcare expenditures by sex among adults with diabetes

Variables	Incremental Cost	95% Confidence Interval	*P*-value
Out-of-pocket expenses			
Prescription	156	87–225	<0.001***
Office-Based	53	24–83	<0.001***
Inpatient	−0.36	−21–20	0.972
Outpatient	12	1.5–23	0.026*
ER visit	−3	−8–2	0.285
Dental	19	−7–46	0.156
Home healthcare	9	−1–20	0.080
Others	19	7–30	0.001**
Pooled	242	134–350	<0.001***
Total direct expenditures			
Prescription	184	50–318	0.007**
Office-Based	2	−153–158	0.975
Inpatient	−213	−563–136	0.232
Outpatient	45	−73–165	0.454
ER visit	−23	−58–11	0.180
Dental	25	−17–68	0.245
Home healthcare	59	2–116	0.041*
Others	−0.86	−21–20	0.936
Pooled	232	−300–765	0.392

Reported as dollars in 2014. *Level of significance *p* < 0.05; ** level of significance *p* < 0.01; ***level of significance *p* < 0.001. Reference group: men with diabetes

Primary outcome variables in this regression model are out-of-pocket and total direct expenditures controlling for age, sex, race/ethnicity, marital status, education, health insurance, MSA, census region, income, a number of comorbid conditions (hypertension, CVD, stroke, emphysema, joint pain, arthritis and asthma) and time trend

## Discussion

In this sample of adults with diabetes, women with diabetes had statistically higher healthcare expenditures compared to men, after controlling for relevant confounding factors. In unadjusted analyses, women had significantly higher mean OOP and total expenditures for prescription services compared to men. In addition, women had higher total direct expenditures for home healthcare in comparison to men. After adjusting for sociodemographic characteristics, comorbidities, and time, these statistically significant differences persisted. In adjusted analyses, women had higher incremental OOP costs for prescription medications, office-based visits, and other services, and also had significantly higher total direct expenditures for prescriptions and home healthcare services compared to men with diabetes. While higher OOP and total direct expenditures for women might reflect higher utilization, it is imperative to determine best practices for keeping costs low and reducing the financial burden of diabetes care for women.

Prior evidence has demonstrated higher healthcare expenditures in women with diabetes. In this sample, women with diabetes were found to have significantly higher expenditures compared to men, particularly for healthcare services including office-based visits, home healthcare, and prescriptions. Zhou et al. found women with diabetes to have higher estimated annual medical spending and excess lifetime incremental expenses compared to men with diabetes [1]. Similarly, in a cohort of adults with diabetes in the United States, followed from 1997 to 2006, women had higher healthcare expenditures than men [32]. For example, NHW women with a BMI > 40 kg/m had lifetime healthcare expenditures in the amount of $185,609, and NHW women with a BMI between 18.5 and 24.9 kg/m had lifetime healthcare expenditures that totaled $183,704 compared to men [32]. More evidence is needed to determine exactly why women with diabetes have higher healthcare expenditures compared to men.

The findings of this study are important because they show women have higher incremental costs associated with health services identified as instrumental for diabetes management: prescriptions, office-based visits, and home healthcare services. Prescription medications are needed, not only for managing and controlling diabetes, but also to slow the progression of adverse complications, which can contribute to expenditures when diagnosed. Evidence suggests, for example, that when women have higher healthcare expenses, they often neglect their own care and basic material securities because of medication costs [33, 34]. In a study to identify problems associated with OOP costs among older adults with diabetes, Piette et al. [34] found women more likely to reduce their use of prescribed medications and spend less on basic needs when met with the difficult choice of paying for prescriptions. In addition to prescriptions, office-based visits with providers and procedures are necessary for routine follow-up and clinical assessments (i.e., glycosylated hemoglobin A1c and lipid measurements, along with monitoring of blood pressure, etc.). Furthermore, home healthcare is often required to provide diabetes education (i.e., use of glucometer, examinations of feet, etc.) or to deliver needed equipment and supplies (i.e., glucometers, test strips, lancing devices).

Given the individual and collective importance of various healthcare services in managing diabetes, it is important to determine best practices for lowering associated costs, which will likely lead to improvements in health outcomes among adults with diabetes. In this regard, interventions could be useful directly, to obtain a better understanding of diabetes-related differences in health outcomes by sex and inform policy, clinical practice, and future research, and indirectly, to help minimize sex-specific differences in outcomes, including costs. Interventions that focus on sex differences in diabetes education, self-management, and lifestyle behaviors such as medication adherence, independently or in concert, are critical for helping to improve overall health and outcomes and reducing the need for additional healthcare services that could result in higher costs. These interventions could also stress the importance of preventive services that can be beneficial in preventing or reducing the onset of comorbid conditions and diabetes-related complications, which can lead to higher differential costs. Cost-benefit analyses could be included as a component in the intervention to address concerns related to diabetes care costs and brainstorm approaches for accessing resources to help minimize these expenses. Provider offices, patient homes, and community settings are all possible locations for delivering the interventions and should be determined based on the needs of the adult participants. In addition, as technology continues to advance, interventions that utilize telehealth and telemonitoring are essential in helping to reduce expenditures associated with seeking health services by reducing the need, for example, for excessive outpatient and emergency room visitations.

There are several limitations that must be mentioned. First, the research design for this study is cross-sectional, which limits the ability to infer cause and effect relationships. Second, there are potential confounders that were not controlled for including healthcare utilization and access to care, self-management practices including medication adherence, diabetes knowledge, and social support. Additionally, we did not account for type or duration of diabetes nor the types and quantities of medications prescribed. Third, the findings of this study are based on self-reported data; therefore, the possibility for under- and/or over-reporting and estimating is possible. However, self-reported diabetes has been shown to be reliable with 98% agreement with a medical record [35]. Fourth, these analyses focus on OOP and total direct costs and do not consider indirect costs, which might differ between men and women with diabetes.

## Conclusions

The results of this study are important and provide new information about differences in healthcare expenditures between men and women with diabetes. In this nationally representative sample of adults with diabetes, women were found to have significantly higher healthcare expenditures for care compared to men, after adjusting for confounding factors. These findings suggest a need for policies that support the prevention of diabetes and its associated complications and focus on the reduction of OOP and total direct expenditures associated with inadequately controlling and managing diabetes in adults. Also, clinicians must ensure clinical guidelines are followed to reduce the development of comorbid conditions and delay the onset of adverse complications, both of which can influence costs. In addition, providers should proactively assist patients by facilitating their access to resources such as medication assistance programs and patient navigators, who consistently and frequently work directly with patients to determine ways for reducing costs associated with care. Finally, the literature evaluating sex differences in healthcare expenditures among adults with diabetes is limited. Further research is needed to understand the drivers of these differences and to identify potential causes associated with higher costs in women with diabetes.

## Abbreviations

ADA: American Diabetes Association
AHRQ: Agency for Healthcare Research and Quality
CI: Confidence Interval
CVD: Cardiovascular Disease
GLM: Generalized Linear Model
MEPS-HC: Medical Expenditure Panel Survey Household Component
MEPS: Medical Expenditure Panel Survey
MSA: Metropolitan Statistical Area
NAMCS: National Ambulatory Medical Care Survey
NHAMCS: National Hospital Ambulatory Medical Care Survey
NHB: Non-Hispanic Black
NHIS: National Health Interview Survey
NHW: Non-Hispanic White
OLS: Ordinary Least Squares
OOP: Out-Of-Pocket
U.S.: United States
VIF: Variance Inflation Factor
